# Safety and efficacy of oral administrated cepharanthine in non-hospitalized, asymptomatic or mild COVID-19 patients: a Double-blind, randomized, placebo-controlled trial: Author detials

**DOI:** 10.1038/s41598-024-75891-3

**Published:** 2025-01-31

**Authors:** Jianyi Wei, Shupeng Liu, Yuexiang Bian, Lei Li, Biyun Qian, Zixuan Shen, Yan Zhang, Adila Abuduaini, Fuchen Dong, Xin Zhang, Jinhui Li, Yongpei Yu, Weituo Zhang, Jun Wang, Wei Zhai, Qixiang Song, Yu Zheng, Weihua Pan, Lanlan Yu, Qimin Zhan, Ning Zhang, Junhua Zheng, Shuming Pan, Chen Yao, Hai Li

**Affiliations:** 1 Department of Gastroenterology, Renji Hospital, Shanghai Jiao Tong University School of Medicine; NHC Key Laboratory of Digestive Diseases (Renji Hospital, Shanghai Jiaotong University School of Medicine), 1630 Dong Fang Road, Shanghai, 200127 China; 2 Department of Emergency, Xinhua Hospital, Shanghai Jiao Tong University School of Medicine, Yangpu District, Shanghai, China; 3 Peking University Clinical Research Institute, Peking University First Hospital, Beijing, 100083 China; 4 Clinical Research Center, Shanghai Jiao Tong University School of Medicine, Shanghai, China; 5 Department of Interventional Oncology, Renji Hospital, Shanghai Jiao Tong University School of Medicine, No. 160 Pujian Rd, Pudong, Shanghai, 200127 China; 6 Department of Urology, Renji Hospital, Shanghai Jiao Tong University School of Medicine, Shanghai, China; 7 Department of Respiratory Medicine, Renji Hospital, Shanghai Jiao Tong University School of Medicine, Shanghai, China; 8 Department of Otorhinolaryngology-Head & Neck Surgery, Xinhua Hospital, Shanghai Jiao Tong University School of Medicine, Shanghai, China; 9 Peking University - Yunnan Baiyao International Medical Research Center, Beijing, China; 10 Hongqiao International Institute of Medicine, Shanghai Tongren Hospital/School of Public Health, Shanghai Jiao Tong University School of Medicine, Shanghai, China; 11 Department of Pediatric Surgery, Xinhua Hospital, Shanghai Jiao Tong University School of Medicine, Shanghai, China

**Keywords:** Cepharanthine, COVID-19, Effectiveness, Safety, SARS-CoV-2, Randomized controlled trials, Viral infection, Epidemiology

## Abstract

Cepharanthine (CEP) is a natural remedy that potently inhibits SARS-CoV-2 activity both in vitro and in vivo. To evaluate the efficacy and safety of CEP compared with placebo in adults with asymptomatic or mild coronavirus disease 2019 (COVID-19), we conducted a proof-of-concept, double-blind, randomized, placebo-controlled trial. Patients were randomized to receive 120 mg/day of CEP, 60 mg/day CEP or placebo for 5 days. Main outcome was the time from randomization to negative nasopharyngeal swab and safety. Among 262 randomized participants, 188 completed the trial among group of 120 mg/day CEP (*n* = 65), 60 mg/day CEP (*n* = 68) and placebo (*n* = 55). Neither 120 mg/day or 60 mg/day CEP shortened the time to negative significantly compared with placebo. However, 60 mg/day CEP showed a slight trend (difference=-0.77 days, hazard ratio (HR) = 1.40, 95% CI 0.97–2.01, *p* = 0.072). In analysis of participants with good medication compliance, 60 mg/day CEP significantly shortened the time to negative (difference=-0.87 days, HR = 1.56, 95% CI 1.03–2.37, *p* = 0.035). Adverse events were not different among the three groups, and no serious adverse events occurred. In conclusion, treatment of asymptomatic or mild Covid-19 with 120 mg/day or 60 mg/day did not shorten the time to negative significantly. However, 60 mg/day CEP showed a slight trend which needs future confirmatory trials to validate. (NCT05398705).

**Supplementary Information:**

The online version contains supplementary material available at 10.1038/s41598-024-75891-3.

## Introduction

Coronavirus disease 2019 (COVID-19), caused by severe acute respiratory syndrome coronavirus 2 (SARS-CoV-2), has caused over 700 million confirmed cases and has placed a heavy burden on global health^1^. SARS-CoV-2 has shown a remarkably fast evolutionary rate, and five major variants of SARS-CoV-2 (Alpha, Beta, Gamma, Delta, and Omicron) have emerged to date^2^. In view of the large number of confirmed cases of COVID-19 across rich and poor regions^3^, the rapid evolution of SARS-CoV-2 and limited and expensive antiviral drugs, it is urgent to identify effective, widely available and inexpensive drugs with broad-spectrum antiviral ability against SARS-CoV-2.

Drug repurposing is a feasible approach in a pandemic. Cepharanthine (CEP) is a biscoclaurine alkaloid extracted from Stephania Cepharantha Hayata that has been used to treat various diseases (including leukopenia, alopecia and snake bites)^4,5^. CEP was identified as a potential SARS-CoV-2 antiviral drug via high-throughput screening in a recent interactome-informed drug repurposing study^6^. It has been reported that CEP is capable of blocking viral entry by binding to the SARS-CoV-2 S protein to interfere with the interaction of the S protein and its receptor angiotensin-converting enzyme 2 (ACE-2)^7–10^. Moreover, SARS-CoV-2 nonstructural protein 13 (Nsp13) is important for the replication of the viral genome and responsible for viral viability^11^. CEP displays antiviral activity in inhibiting Nsp13 ATPase (helicase), thus inhibiting viral replication^12^. CEP shows potential antiviral activities against SARS-CoV-2 both in vitro and in vivo^13,14^. In addition, CEP, a natural alkaloid that has been used for a long time in the treatment of various diseases, shows no significant side effects in patients^15^. Given the well-established safety profile and significant unique antiviral effect of CEP, repurposing inexpensive and easily available CEP as an antiviral drug for the treatment of COVID-19 is of great potential.

However, information about the efficacy and safety of CEP in inhibiting SARS-CoV-2 replication to treat COVID-19 is still lacking, and clinical trials are urgently needed. Thus, during the epidemic of COVID-19 in Shanghai, China, we started a randomized, double-blind and placebo-controlled clinical trial to evaluate the safety and efficacy of CEP in inhibiting SARS-CoV-2. Given the high transmissibility but low pathogenicity of Omicron variant, the dominant SARS-CoV-2 variant at present, most of SARS-CoV-2-infected patients are asymptomatic or mild^16^. Therefore, patients enrolled in this study were non-hospitalized adults with asymptomatic and mild COVID-19, and as the key index to evaluate the efficacy, the time from randomization to negative nasopharyngeal swab was the primary endpoint in this study.

## Methods

### Trial design and oversight

This was a proof-of-concept, double-blind, stratified randomized, parallel, placebo-controlled trial. The study enrolled non-hospitalized patients with asymptomatic and mild COVID-19 and a confirmed positive polymerase chain reaction (PCR) test for SARS-CoV-2 infection. The trial protocol is available in ClinicalTrials.gov. (First posted date: 01/06/2022, Registration number: NCT05398705).

The trial protocol was approved by each site’s institutional review board. Informed consent was obtained from each participant in writing. An independent data and safety monitoring committee oversaw participant safety, efficacy, and trial conduct.

### Participants

Participants were recruited between May 31, 2022 and July 24, 2022, from the alternate care site at Shanghai New International Expo Centre, China (managed by Renji Hospital, School of Medicine, Shanghai Jiaotong University) and alternate care site at Shanghai Chongming Fuxing, China (managed by Xinhua Hospital, School of Medicine, Shanghai Jiaotong University). Alternative care sites, also known as Fangcang hospitals, were temporary medical facilities established during the COVID-19 pandemic in China to treat patients in a centralized and quarantine environment. Patients with asymptomatic or mild COVID-19 were admitted to the alternate care sites and discharged after two consecutive negative nucleic acid tests. All participants provided written informed consent. This trial was registered on ClinicalTrials.gov (NCT05398705).

The inclusion criteria for this study included age from 16 to 85 years, confirmed SARS-CoV-2 infection by PCR, SARS-CoV-2 infection for less than 5 days prior to randomization, a diagnosis of asymptomatic or mild COVID-19 according to WHO guidelines^1^, and signed informed consent.

Key exclusion criteria included pneumonia or severe COVID-19^17^, acute exacerbation of chronic underlying diseases, and pregnancy or lactation. Asymptomatic COVID-19 was defined as a positive SARS-CoV-2 test without symptoms^18^. Mild COVID-19 was defined as confirmed COVID-19 infection complicated by mild symptoms, including fever, cough, or changes in taste or smell, without evidence of dyspnea or pneumonia on imaging^18^. Additional information of patients is available in the Supplementary materials.

### Blinding and masking

Firstly, the pharmaceutical factory (Yunnan Baiyao Group Co., Ltd) produced placebo tablets identical in appearance, texture, smell, and taste to CEP tablets. Then, a third-party platform (Ali Health platform, https://www.mashangfangxin.com/) assigned a unique code to each medication (i.e., each participant’s 5-day medication with 15 doses totally, 2 CEP tablets/dose for 120 mg/day CEP group, 1 CEP tablet and 1 placebo tablet/dose for 60 mg/day CEP group, 2 placebo tablets/dose for placebo group) based on drug information, then encrypted this code, and uploaded the medication blinding to the central randomization system. Finally, the pharmaceutical factory packaged each medication uniformly and affixed the corresponding encrypted code onto the packaging. All people involved in the conduct of the clinical trial and participants were masked to treatment allocation.

### Randomization

**Figure 1:**
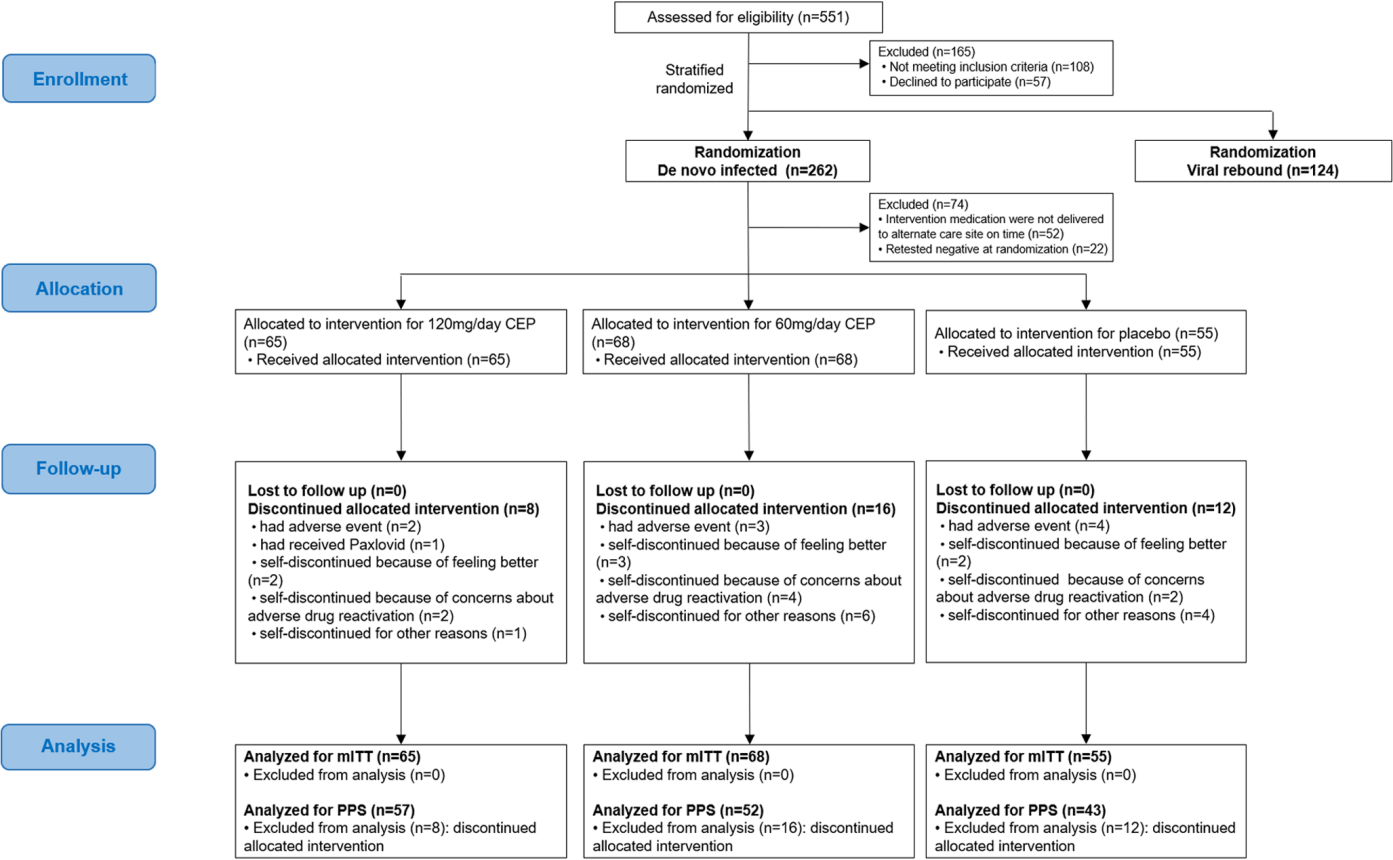
Randomization, treatment assignments, and follow-up. The figure shows that patients were recruited through May 31, 2022, to July 24, 2022, from two alternate care sites in Shanghai, China, and underwent stratified randomization according to de novo infection or viral rebound of SARS-CoV-2. Treatment assignments and follow-up were conducted.

Stratified randomization was performed according to de novo SARS-CoV-2 infection and viral rebound (Details information and all results of viral rebound of SARS-CoV-2 patients are in supplementary materials, the results in the following text refer specifically to the de novo SARS-CoV-2 infected patients). Participants were 1:1:1 assigned using a random number generated by a centralized randomization system provided by Clinical Information Management Suite (CIMS) Medical Technology Company (Chengdu, China) to 60 mg/day CEP, 120 mg/day CEP or matching placebo group (Fig. 1). Researchers, patients, caregivers and statisticians were masked to allocation, and a separate unblinded data monitoring committee evaluated safety throughout this study.

### Interventions

Participants received 40 mg of CEP, 20 mg of CEP or matched placebo orally every 8 h for 5 days (15 doses total) or until negative conversion. Researchers dispensed the 5-day medication or placebo to participants on the day of enrollment. Subsequently, dedicated researchers visited participants three times a day to inquire about medication adherence and adverse reactions.

CEP and matching placebo were manufactured by Yun Nan Bai Yao Pharmaceutical Group Inc. (Z20026797), with packaging for the matching placebo identical to that of the active drug. All patients at alternate care sites received standard medical treatment according to the Scheme for Diagnosis and Treatment of 2019 Novel Coronavirus Pneumonia (The 9th Trial Edition) from the Health Commission of China, including bed rest, adequate energy and nutrition, plenty of water, traditional Chinese medicine treatment, etc^19^.

### Outcome measures

The primary outcome of efficacy was the time from randomization to negative nasopharyngeal swab, which was defined as the duration from randomization to negative conversion (the first of two consecutive negative nasopharyngeal swabs tested by PCR, with a Ct value > 35 for the ORF1ab and N genes). The secondary outcomes were the proportion of patients who progressed to pneumonia or severe COVID-19 and the proportion of patients who were SARS-CoV-2 positive after negative nasopharyngeal swab.

Safety endpoints included adverse events, serious adverse events, and any adverse events that contributed to discontinuation of the study intervention that occurred during the treatment period and during the follow-up periods. Reported adverse events were coded according to the Medical Dictionary for Regulatory Activities (MedDRA), version 25.0. The safety analysis population included all patients who received at least one dose of the study intervention drug. The incidence data for each treatment group were analyzed within the safety analysis population.

### Trial procedures

Screening and eligibility confirmation occurred at alternate care sites. A positive SARS-CoV-2 PCR test was verified prior to randomization and was retested at randomization. At screening, demographic information was collected which included eligibility criteria, medical history, concomitant medications, symptom reporting and vaccination history of SARS-CoV-2.

Researchers enrolled the participants and registered them in the central randomization system, then the system generated a subject ID and random number to perform randomization, after that, the system gave the encrypted code of medication. On the day of randomization, researchers scanned the same encrypted code on the medication packaging, and the third-party drug distribution platform verified this operation, then researchers dispensed the 5-day medication to the participant. Shipping and delivery were tracked. Participants must have received the study medication to be include in the analysis. At the alternate care sites, starting from Day1 (the day of randomization), nasopharyngeal swabs were collected for nucleic acid testing on Day 3, 5 and daily thereafter, and the results were recorded. Post-discharge, community staff collected nasopharyngeal swabs for nucleic acid test every day. During the 28-day follow-up (from the time of randomization), all participants underwent standardized procedures. Throughout the alternate care sites, researchers visited participants three times daily, including in-person visits in the morning and afternoon, as well as a telephone visit in the evening. Post discharge, telemedicine follow-ups were conducted weekly. Researchers queried and recoded information of nucleic acid test results, presence of symptoms, improvement or worsening of symptoms, development of pneumonia or severe COVID-19, and any adverse reactions following medication intake.

### Statistical analysis

All eligible patients who received at least one dose of the drug or placebo who also tested positive by nucleic acid test at randomization were included in the modified intention-to-treat (mITT) population, with patients who completed the treatment (had good medical compliance (80-120%)) included in the per-protocol set (PPS). All patients who received at least one dose of the drug or placebo were included in the safety analysis set (SS).

In the original design of this study, the time from randomization to negative nasopharyngeal swab was used as the primary endpoint. The sample size of this study was determined to detect a potential clinical superiority of CEP in time to viral shedding. According to the experimental results of CEP against SARS-CoV-2 virus in vivo and in vitro, we supposed a total of 105 patients in each group would provide 80% power to detect a hazard ratio (HR) of 1.5, which was tested in the Cox proportional hazards model of the time to negative nasopharyngeal swab for CEP versus placebo. The overall probability of an event was 0.9, with a two-sided significance level of α = 0.05, and the ratio of the sample in each group was 1:1:1. Considering the explorative property of this study and that the assumptions in sample size determination were based on limited clinical evidence, this study continued to enroll patients after 315 patients were enrolled when research resources were sufficient.

The analysis for efficacy assessment was performed in both the mITT set and the PPS. The analysis performed in the mITT set was primary analysis, and that performed in the PPS was considered supportive analysis. Continuous variables are presented as the means with standard deviations or medians with interquartile ranges (IQRs), and categorical variables are reported as numbers and percentages. There were no missing data in the mITT set.

Since all patients were quarantined until they tested negative for SARS-CoV-2 infection, there was no censoring for time to negative nasopharyngeal swab due to loss to follow-up in this study. Death was treated as a competitive event of viral shedding. The time to negative nasopharyngeal swab of each group was summarized and the restricted mean survival time (RMST) and corresponding 95% confidence intervals (CI)of each group were estimated. A Cox proportional hazards model was fitted to estimate the HR and 95% CI for the 60 mg/day CEP group and 120 mg/day CEP group compared with the control group, and the reported p value was evaluated by the Cox proportional hazards model. In this model, underlying disease (presence or absence), age (> 60 years or ≤ 60 years), sex (male or female), symptoms on admission (symptomatic or asymptomatic) and days from the first nucleic acid test to randomization were included as prespecified adjusted variables. In addition, Prespecified subgroup analyses of primary and secondary endpoints were conducted, and 95% CIs were provided to evaluate whether the treatment effect varied according to age, sex, symptoms (asymptomatic or mild), days from first nucleic acid test to randomization, vaccination, and high-risk factors for progression to severe COVID-19 (including age ≥ 60 years, smoking, obesity, and underlying clinical conditions)^20–22^. The statistical analysis plan (SAP) is attached in the Supplementary materials.

The CONSORT reporting guidelines were attached in the supplementary CONSORT checklist^23^.

## Results

### Study population

Between May 31 and July 24, 2022, a total of 551 patients were screened for inclusion at two sites in Shanghai, China, 262 de novo SARS-CoV-2 infected patients underwent randomization (Only de novo infection participants were reported in the text, results of viral rebound participants see Supplementary materials). Among the randomized patients, 52 patients at Renji Hospital’s alternate care site did not receive the intervention drugs between June 1, 2022, and June 3, 2022, in addition, 22 were confirmed to be SARS-CoV-2 negative by nucleic acid test at randomization (see Supplementary materials for detailed reasons). Therefore, the modified intention-to-treat (mITT) set rather than the intention-to-treat (ITT) set was selected for efficacy analysis in this study. Consequently, 188 patients from group of 120 mg/day CEP (*n* = 65), 60 mg/day CEP (*n* = 68) and placebo (*n* = 55) were included in the mITT set. The overall medication compliance was 85.98%, and 152 patients achieved a compliance rate of 80% or more, who were included in the per-protocol (PPS) analysis. The number of patients in the PPS of the 120 mg/day, 60 mg/day and placebo groups was 57, 52 and 43, respectively [Fig. 1].

In the mITT population, the median ages in the 120 mg/day CEP, 60 mg/day CEP and placebo groups were 41.00 years [31.00, 54.00], 35.50 years [26.50, 49.25] and 43.00 years [31.50, 51.50], respectively. Patients with mild COVID-19 who had symptoms before enrollment in the three groups were 67.7% (44/65), 61.8% (42/68), and 60.0% (33/55), and the patients still had symptoms at enrollment were 29.2% (19/65), 23.5% (16/68) and 30.9% (17/55), respectively. The percentages of patients who were at high risk of developing severe COVID-19 were 53.8% (35/65), 53.7% (36/68) and 49.1% (27/55) in the three groups, of which the percentages of patients with chronic underlying diseases were 20.0% (13/65), 20.6% (14/68) and 16.4% (9/55), respectively [Table 1].

**Table 1:** Demographic and clinical characteristics of the patients (modified intention-to-treat population).

Characteristic	No.(%)				
	Overall	120 mg/day CEP	60 mg/day CEP	Placebo	*P* value
**No.**	188	65	68	55	
**Age**,** median (IQR)**,** y**	40.00 [29.00, 52.25]	41.00 [31.00, 54.00]	35.50 [26.50, 49.25]	43.00 [31.50, 51.50]	0.272
**>60 y (%)**	21 (11.2)	9 (13.8)	7 (10.3)	5 (9.1)	0.706
**Sex (%)**					0.068
**female**	74 (39.4)	20 (30.8)	34 (50.0)	20 (36.4)	
**male**	114 (60.6)	45 (69.2)	34 (50.0)	35 (63.6)	
**Symptom type of patients (%)**					
**Symptomatic**	119 (63.3)	44 (67.7)	42 (61.8)	33 (60.0)	0.648
**Had symptoms at enrolment**	52 (27.7)	19 (29.2)	16 (23.5)	17 (30.9)	0.646
**Fever**	5 (2.7)	3 (4.6)	0 (0.0)	2 (3.6)	0.231
**Cough**	48 (25.5)	17 (26.2)	16 (23.5)	15 (27.3)	0.912
**Asymptomatic**	69 (36.7)	21 (32.3)	26 (38.2)	22 (40.0)	
**Days from first nucleic acid test to randomization (%)**					0.755
**≤ 3 days**	101 (53.7)	33 (50.8)	39 (57.4)	29 (52.7)	
**4–5 days**	87 (46.3)	32 (49.2)	29 (42.6)	26 (47.3)	
**Vaccine (%)**					0.149
**Not vaccinated**	32 (17.0)	8 (12.3)	14 (20.6)	10 (18.2)	
**Vaccinated**,** 1–2 doses**	67(35.7)	18 (27.7)	28 (41.2)	21 (38.2)	
**Vaccinated**,** 3 doses**	89 (47.3)	39 (60.0)	26 (38.2)	24 (43.6)	
**Have Chinese traditional medicine (%)**	134 (71.3%)	50 (76.9)	44 (64.7)	40 (72.7)	0.301
Patients at high risk of developing severe COVID-19 (%)*	98 (52.4)	35 (53.8)	36 (53.7)	27 (49.1)	0.868
**With underlying chronic disease**	36 (19.1)	13 (20.0)	14 (20.6)	9 (16.4)	
**Age > 60 y**	21 (11.2)	9 (13.8)	7 (10.3)	5 (9.1)	
**BMI > 25 kg/m** ^**2**^	47 (25.1)	16 (24.6)	17 (25.4)	14 (25.5)	
**Cigarette smoking**	50 (26.6)	18 (27.7)	18 (26.5)	14 (25.5)	

*Patients at risk of developing severe COVID-19 were defined as have ≥ 1 of following: >60 year of age; chronic underlying disease; BMI > 25 kg/m^2^; cigarette smoking.

### Primary outcome

**Figure 2:**
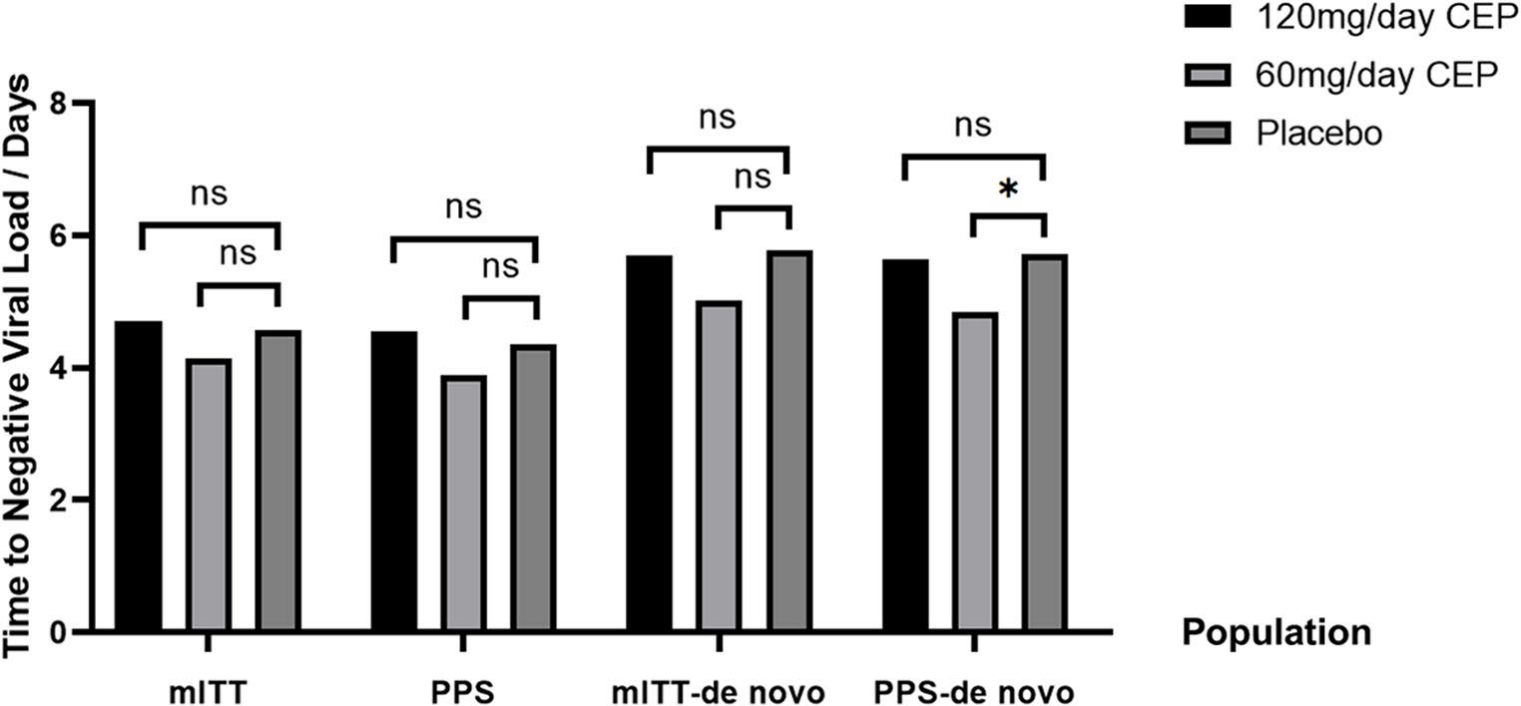
Time to negative viral load in three groups of different populations. The bar plot shows the primary endpoint of time from randomization to negative nasopharyngeal swab; from left to right are the times of the 120 mg/day CEP, 60 mg/day CEP and placebo groups in the mITT set, patients with good medication compliance (PPS), de novo infection patients in the mITT set, de novo infection patients in the PPS. P values were reported by the Cox proportional hazards model which survival function estimation method was the Eforn method, and the adjusted variables included group, age (> 60 years or ≤ 60 years), gender, symptom status (had symptoms at enrolment or not), with or without underlying disease and the days from first nucleic acid test to randomization. An asterisk (*) indicates a significant difference between the two groups in the Cox regression analysis results (p-value < 0.05), while “ns” indicates no significant difference.

In the mITT set, the time to negative nasopharyngeal swab in the 120 mg/day CEP, 60 mg/day CEP and placebo groups was 5.71 (95% CI 5.00 to 6.42), 5.01 (95% CI 4.37 to 5.66), and 5.78 (95% CI 4.88 to 6.41) days, respectively. Compared with placebo, there was no significant difference in the time to negative nasopharyngeal swab for the 120 mg/day CEP group (RMST difference=-0.07 days, HR = 1.10, 95% CI 0.76 to 1.60, *p* = 0.606), while there was a slight trend of a shorter time to negative nasopharyngeal swab in the 60 mg/day CEP group (RMST difference=-0.77, HR = 1.40, 95% CI 0.97 to 2.01, *p* = 0.072) [Table 2] [Fig. 2].

**Table 2:** Primary and secondary outcomes in three groups.

Group, No. (%)	mITT			PPS		
	120 mg/day CEP	60 mg/day CEP	Placebo	120 mg/day CEP	60 mg/day CEP	Placebo
**No.**	65	68	55	57	52	43
**Primary outcome**,** time to negative**						
**Outcome event (%)**	65 (100)	68 (100)	55 (100)	57 (100)	52 (100)	43 (100)
**RMST (95%CI)**, days*	5.71 (5.00,6.42)	5.01 (4.37, 5.66)	5.78 (4.88, 6.68)	5.63 (4.86, 6.41)	4.85 (4.12, 5.58)	5.72 (4.69, 6.75)
**Difference of RMST ** **(95%CI)**	-0.07 (-1.12, 1.07)	-0.77(-1.88, 0.34)	-	-0.09(-1.38, 1.20)	-0.87(-2.14, 0.39)	-
**Hazard Ratio (95%CI)†**	1.10 (0.76, 1.60)	1.40 (0.97, 2.01)	-	1.11 (0.73, 1.68)	1.56 (1.03,2.37)	-
**P value††**	0.606	0.072	-	0.619	0.035	-
**Secondary outcomes**						
**Patients developed to pneumonia or severe COVID-19 — no.(%)**	0(0)	0(0)	0(0)	0(0)	0(0)	0(0)
**SARS-CoV-2 returned to positive after turning negative — no.(%)**	2 (3.1)	1 (1.5)	3 (5.5)	2 (3.5)	1 (1.9)	3 (7.0)
**Hospitalization for COVID-19 — no.(%)**	0(0)	0(0)	0(0)	0(0)	0(0)	0(0)

* Time to negative was the time from randomization to negative nasopharyngeal swab, it was evaluated by RMST.

† Hazard ratio evaluated by Cox proportional hazards model was primary analysis, adjusted for underlying chronic disease, age (> 60 year or ≤ 60 year), gender, symptoms, days from first nucleic acid test to randomization.

†† p value was reported from adjusted cox regression.

In the PPS, the time to negative nasopharyngeal swab in the 60 mg/day CEP and placebo groups was 4.85 days (95% CI 4.12 to 5.58) and 5.72 days (95% CI 4.69 to 5.75), respectively. The 60 mg/day of CEP significantly shortened the time by 0.87 days compared with placebo (RMST difference= -0.87, HR = 1.56, 95% CI 1.03 to 2.37, *p* = 0.035), while 120 mg/day did not show efficacy (RMST difference=-0.09, HR = 1.11, 95% CI 0.73 to 1.68, *p* = 0.619) [Table 2] [Fig. 2].

**Figure 3:**
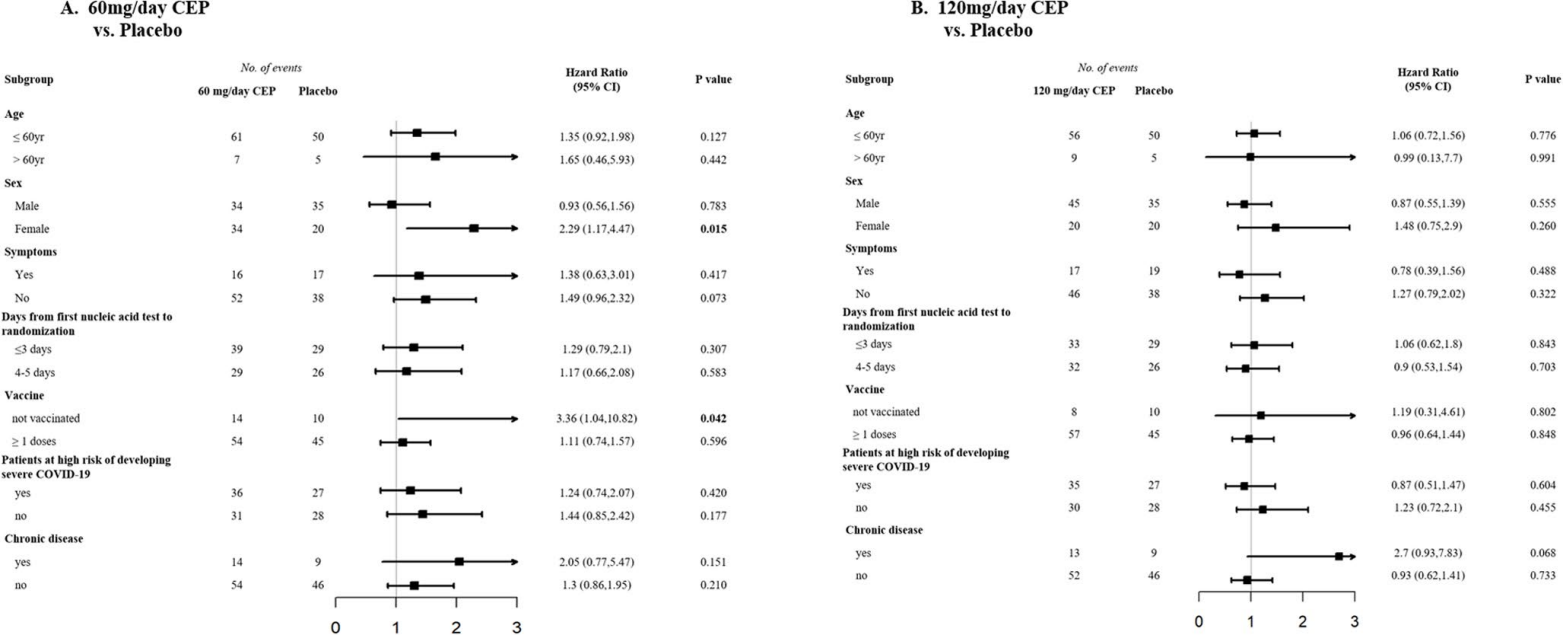
Subgroup analysis of the difference in the time to negative nasopharyngeal swab compared to placebo (mITT). Panel **A** shows the subgroup analysis of between patients who received 60 mg/day of CEP and those who received placebo. Panel **B** shows the subgroup analysis between patients who received 120 mg/day of CEP and those who received placebo.

In the subgroup analysis among patients in the mITT set, we observed a benefit with 60 mg/day of CEP compared with placebo among patients who were not vaccinated (RMST difference = -3.43 days, HR = 3.36, 95% CI 1.04 to 10.82) and among female patients (RMST difference = -2.37 days, HR = 2.29, 95% CI 1.17 to 4.47) [Fig. 3].

### Secondary outcome

No patients in the mITT set progressed to severe COVID-19 during the 28-day follow-up. Among mITT patients, the proportions who were SARS-CoV-2 positive after negative nasopharyngeal swabs were 3.1% (2/65), 1.5% (1/68) and 5.5% (3/55) in the 120 mg/day CEP, 60 mg/day CEP and placebo groups, respectively, with no significant differences observed [Table 2].

### Adverse events

A total of 317 patients were included in the safety analysis set, and we compared safety endpoints among the 120 mg/day CEP, 60 mg/day CEP and placebo groups. There were 156 adverse events that emerged during the treatment period. The incidence rates of adverse events that emerged during or after the treatment period in the 120 mg/day CEP, 60 mg/day CEP and placebo groups were 33.65% (35/104), 37.72% (43/114) and 35.35% (35/99), respectively. Adverse events were similar in the three groups and included diarrhea, increased bowel movement frequency, drowsiness, dizziness, night sweats and others. No serious adverse events were reported in any safety analysis population. There were 81 adverse events considered by the site investigator to be related to the trial drug, and the incidence rates in the three groups were 24.04% (25/104), 23.68% (27/114), and 20.20% (20/99). The most frequently reported events (affecting at least 1% of patients) were diarrhea (13 (12.50%) in the 120 mg/day CEP group vs. 9 (7.89%) in the 60 mg/day CEP group vs. 9 (9.09%) in the placebo group), drowsiness (6 (5.77%) in the 120 mg/day CEP group vs. 10 (8.77%) in the 60 mg/day CEP group vs. 7 (7.07%) in the placebo group), night sweats (6 (5.77%) in the 120 mg/day CEP group vs. 9 (7.89%) in the 60 mg/day CEP group vs. 1 (1.01%) in the placebo group), and dizziness (3 (2.88%) in the 120 mg/day CEP group vs. 0 (0%) in the 60 mg/day CEP group vs. 1 (1.01%) in the placebo group). During the treatment period, there were no grade 3 or 4 adverse events, and all events were mild to moderate (grade 1 or 2) and were resolved at the time of this analysis [Table 3].

**Table 3:** Summary of adverse events, serious adverse events and adverse events leading to discontinuation through day 28 (Safety Analysis Population*).

Adverse Event Category	120 mg/day CEP (*N* = 104)	60 mg/day CEP (*N* = 114)	Placebo (*N* = 99)
**Events that emerged during treatment period**			
No. of adverse events	48	66	42
**Patients with adverse events — no. (%)**			
Any adverse event	35(33.65)	43(37.72)	35(35.35)
Serious adverse event	0(0)	0(0)	0(0)
Maximum grade 3 or 4 adverse event	0(0)	0(0)	0(0)
Maximum grade 5 adverse event	0(0)	0(0)	0(0)
Discontinued drug or placebo because of adverse event	2(1.92)	4(3.51)	6(6.06)
Had dose reduction or temporary discontinuation owing to adverse event	1(0.96)	1(0.88)	1(1.01)
**Adverse Event Type — no. (%)**			
Diarrhea	19 (18.27)	22 (19.30)	15 (15.15)
Increased bowel frequency	9(8.65)	15 (13.16)	10 (10.10)
Drowsiness	6 (5.77)	10 (8.77)	7 (7.07)
Dizzy	3 (2.88)	0 (0)	1 (1.01)
Night sweats	6 (5.77)	9 (7.89)	1 (1.01)
Others	5 (4.81)	10 (8.77)	8 (8.08)
**Events considered to be related to drug or placebo**			
No. of adverse events	29	31	21
**Patients with adverse events — no. (%)**			
Any adverse event	25(24.04)	27(23.68)	20(20.20)
Serious adverse event	0(0)	0(0)	0(0)
Maximum grade 3 or 4 adverse event	0(0)	0(0)	0(0)
Maximum grade 5 adverse event	0(0)	0(0)	0(0)
Discontinued drug or placebo because of adverse event	2(1.92)	3(2.63)	4(4.04)
Had dose reduction or temporary discontinuation owing to adverse event	0(0)	1(0.88)	1(1.01)
**Adverse Event Type — no. (%)**			
Diarrhea	13 (12.50)	9 (7.89)	9 (9.09)
Drowsiness	6 (5.77)	10 (8.77)	7 (7.07)
Night sweats	6 (5.77)	9 (7.89)	1 (1.01)
Dizzy	3 (2.88)	0 (0)	1 (1.01)
Others	1 (0.96)	3 (2.63)	3 (3.03)

* Shown are data for all patients who received at least one dose of drug or placebo.

## Discussion

This study is the first randomized placebo-controlled trial to evaluate the safety and efficacy of oral administration of CEP for asymptomatic or mild COVID-19 patients at alternate care sites in China. We explored the safety and efficacy of viral clearance of two different doses of CEP in treating patients with asymptomatic or mild COVID-19. The results showed that the side effects of both 5-day oral administration of 60 mg/day of CEP and 120 mg/day of CEP were generally mild and without safety concerns. In the mITT analysis, neither the 120 mg/day nor the 60 mg/day CEP demonstrated significant effects in shortening the time to negative swab, with only the 60 mg/day CEP showing slight trends. However, it is noteworthy that in the PPS analysis, the time to negative swab was significantly shorter in the 60 mg/day CEP group compared to the placebo group.

Although 60 mg/day of CEP significantly shortened the time to negative swabs in the PPS (HR = 1.56, 95% CI 1.03 to 2.37, *p* = 0.035), it did not achieve a significant difference in the mITT set (HR = 1.40, 95% CI 0.97 to 2.01, *p* = 0.072). Therefore, the efficacy of 60 mg/day of CEP in the PPS should be carefully explained. The possible reason may be the limited sample size. The number of de novo infected patients who underwent randomization was 262, which did not reach the designated sample size of 315 patients, and this study may not have had enough power to detect a significant difference. Based on the hazard ratio of this study, a sufficient sample size may be required for further validation trials.

As a natural alkaloid with multiple targets^5^, CEP has anti-SARS-CoV-2, antioxidant (scavenging free radicals), cell membrane stabilization, drug efflux transporter inhibition, vasodilatation and other properties. However, the specific dose of CEP required to activate each target and the interactions between the targets are not completely clear. In this study, when the dose of CEP was increased to 120 mg/day, it did not show an efficacy in shortening the time to negative nasopharyngeal swab (neither in the mITT set nor in the PPS). Clearance of viruses in patients is determined by the speed of virus replication and the speed of the immune system in eliminating the virus. The possible reason is that in addition to the direct antiviral effect against SARS-CoV-2 ^13,14^, 120 mg/day of CEP may inhibit the ability of the immune system to clear SARS-CoV-2. It has been reported that 10 mg/kg of CEP in mice can not only inhibit the replication of SARS-CoV-2^14^ but also inhibit the NF-κB pathway and reduce the release of cytokines such as TNF-α, IL-1β and IL-6^24^. In addition, CEP inhibits neutrophils^25^, inhibits dendritic cells^26^, stimulates T cells^27^, etc. According to this study, 60 mg/day of CEP significantly shortened the time to negative results among patients with good compliance. We speculate that at a dose of 60 mg/day, CEP dose not activate the target of inhibiting the immune system, while a dose of 120 mg/day of CEP could activate the target. As a result, 120 mg/day of CEP dose not shorten the time to negative swabs.

As a natural alkaloid, CEP has been used to treat various diseases for a long time and has been proven to have good safety^4,5^, which is also shown in the treatment of asymptomatic and mild COVID-19. In this study, no serious adverse events occurred in the 120 mg/day CEP, 60 mg/day CEP or placebo group. The incidence of adverse events in the three groups was similar, and the most common adverse events were diarrhea and drowsiness.

This trial has several strengths. This study is the first randomized clinical trial in the world, to our knowledge, to evaluate the safety and efficacy of oral administration of CEP for non-hospitalized adults with asymptomatic or mild COVID-19, which provided an important data in advancing alternative treatments for COVID-19. Second, this study was designed as a randomized, double-blind, placebo-controlled trial and had high quality, ensuring that the results were scientific. Third, patients selected for this study were non-hospitalized adult patients with asymptomatic or mild COVID-19, which means that the results can be extrapolated. At present, because the Omicron variant has low pathogenicity but high infectivity^2^, asymptomatic and mild infections are the most common and will likely remain in the future. Therefore, the results of this study can be better applied to the overwhelming majority of SARS-CoV-2-infected people.

There are also some limitations of this study. First, the baseline characteristics of the mITT population were not entirely comparable among three assigned groups. Sixty-nine patients did not receive the intervention medicine, and 36 patients had negative nasopharyngeal swabs upon retesting at randomization, therefore, we chose the mITT population as the primary analysis population in our study which caused the imbalance in baseline data among the three groups. We have considered this problem in advance and performed a prespecified adjusted Cox regression analysis in our study, which is a solution to this point. Second, since no patients developed pneumonia or severe COVID-19 during this study, the effect of CEP on preventing severe COVID-19 could not be evaluated. This is reasonable for the characteristics of Omicron variant infection. The Omicron variant is the major epidemic variant of SARS-CoV-2, and its pathogenicity is lower than that of other known variants, which leads to most of SARS-CoV-2-infected patients being asymptomatic or mild^16^, and the rate of developing severe COVID-19 is extremely low^28^. Third, although we considered various factors that could potentially impact outcomes and conducted prespecified subgroup analyses, including vaccination status, we did not collect the data on vaccination dates, thus failing to incorporate the most recent vaccination into our analysis. Lastly, patients enrolled in this study were limited to asymptomatic or mild COVID-19 patients, and the efficacy of CEP for severe COVID-19 remains unknown and needs further study. However, according to the epidemic characteristics of the majority of asymptomatic or mild SARS-CoV-2-infected patients, the number of patients enrolled in this study was appropriate.

## Conclusions

Among asymptomatic or mild COVID-19 patients, oral administration of 120 mg/day of CEP and 60 mg/day of CEP for 5 days is safe. Neither 120 mg/day nor 60 mg/day of CEP shortened the time to negative nasopharyngeal swab. However, 60 mg/day CEP showed a slight trend. Further studies may validate the efficacy of CEP in treating COVID-19 among de novo SARS-CoV-2-infected patients at a dose of 60 mg/day.

## Supplementary Information

Supplementary Material 1.

## Data Availability

All data analyzed in this study could be obtained for non-commercial purposes upon request via email: aclf_group@163.com or haili_17@126.com.
