# Supplementary material for: Safety and efficacy of oral administrated cepharanthine in non-hospitalized, asymptomatic or mild COVID-19 patients: a Double-blind, randomized, placebo-controlled trial: Author detials
Source: Sci Rep. 2025 Jan 31;15:3875. doi: 10.1038/s41598-024-75891-3 (PMC11785718; doi:10.1038/s41598-024-75891-3)
Supplement: Supplementary file 1 — Supplementary Material 1. [file 41598_2024_75891_MOESM1_ESM.docx]

Supplementary Materials for

Safety and Efficacy of Oral administrated Cepharanthine in Non-hospitalized, asymptomatic or mild COVID-19 patients: A Double-blind, Randomized, Placebo-controlled Trial

Jianyi Wei^#1^, Shupeng Liu^#1^, Yuexiang Bian^#1^, Lei Li^#8,^ Biyun Qian^10^, Zixuan Shen^1^, Yan Zhang^1^, , ADila·ABuduaini^1^, Fuchen Dong^1^, Xin Zhang^1^ Jinhui Li^1^, Yongpei Yu^3^, Weituo Zhang^4^ , Jun Wang^5^, Wei Zhai^6^, Qixiang Song^6^, Yu Zheng^7^, , Weihua Pan^11^, Lanlan Yu^3^, Qimin Zhan^9^, Ning Zhang^9^, Junhua Zheng^6^, Shuming Pan^*2^, Chen Yao^*3^, Hai Li^*1^

1. Department of Gastroenterology, Renji Hospital, Shanghai Jiao Tong University School of Medicine; NHC Key Laboratory of Digestive Diseases (Renji Hospital, Shanghai Jiaotong University School of Medicine), 1630 Dong Fang Road, 200127, Shanghai, China 2. Department of Emergency, Xinhua Hospital, Shanghai Jiao Tong University School of Medicine, Yangpu District, Shanghai, China. 3. Peking University Clinical Research Institute, Peking University First Hospital, Beijing, China. 4. Clinical Research Center, Shanghai Jiao Tong University School of Medicine, Shanghai, China. 5. Department of Interventional Oncology, Renji Hospital, Shanghai Jiao Tong University School of Medicine, No. 160 Pujian Rd, Pudong, Shanghai 200127, China. 6. Department of Urology, Renji Hospital, Shanghai Jiao Tong University School of Medicine

7. Department of Respiratory Medicine, Renji Hospital, Shanghai Jiao Tong University School of Medicine, Shanghai, China.

8. Department of Otorhinolaryngology-Head & Neck Surgery, Xinhua Hospital, Shanghai Jiao Tong University School of Medicine, Shanghai, China.

9. Peking University - Yunnan Baiyao International Medical Research Center, Beijing, China

10. Hongqiao International Institute of Medicine, Shanghai Tongren Hospital/School of Public Health, Shanghai Jiao Tong University School of Medicine

11. Department of Pediatric Surgery, Xinhua Hospital, Shanghai Jiao Tong University School of Medicine, Shanghai, China.

Correspondence to: [haili_17@126.com](mailto:haili_17@126.com) or [aclf_group@163.com](mailto:aclf_group@163.com)

**This PDF file includes:**

Materials and Methods

Supplementary Results

Supplementary References

Tables S1 to S4

Figures S1 to S2

Statistical Analysis Plan

**Materials and Methods**

Trial Design and Oversight

This trial was a double-blind, stratified randomization, parallel, placebo-controlled trial. The study has enrolled non-hospitalized patients with asymptomatic or mild COVID-19 and a confirmed positive polymerase chain reaction (PCR) for SARS-CoV-2 infection. The trial protocol and statistical analysis plan are available in the part of "Protocol" and "Statistical Analysis Plan" of the supplementary materials, respectively.

Each site's institutional review board approved the trial protocol. Each participant has written the consent for informed consent. An independent data and safety monitoring committee oversaw participant safety, efficacy, and trial conduct.

Patients

Participants were recruited from May 31st, 2022, to July 24th, 2022, from the alternate care site at Shanghai New International Expo Centre, China (managed by Renji Hospital, School of Medicine, Shanghai Jiaotong University) and alternate care site at Shanghai Chongming Fuxing, China (managed by Xinhua Hospital, School of Medicine, Shanghai Jiaotong University). The trial was ended because the end of this epidemic in Shanghai and the alternate care sites were closed. All participants provided written informed consent. This trial was registered on ClinicalTrials.gov, NCT05398705.

Asymptomatic COVID-19 patients were COVID-19 patients without symptoms^1^. Mild COVID-19 patients were COVID-19 patients with mild symptoms, including fever, cough, or changes in taste or smell, without evidence of dyspnea or pneumonia on imaging^1^.

The inclusion criteria for this study included age from 16 to 85 years, confirmed SARS-CoV-2 infection by PCR (positive nasopharyngeal swab test by PCR: Ct ≤35 for the ORF1ab or nucleocapsid N genes), SARS-CoV-2 infection for less than five days prior to randomization, and signed informed consent. Key exclusion criteria included pneumonia or severe COVID-19^2^, acute exacerbation of chronic underlying diseases, and pregnancy or lactation. Participants received 120 mg CEP/day, 60 mg CEP/day, or matched placebo for 5 days or until negative conversion.

Patients at high risk of developing severe COVID-19 were required to have ≥1 of the following characteristics or comorbidities associated with an increased risk of developing severe COVID-19 illness: ≥60 years of age, body mass index (BMI) >25 kg/m2, cigarette smoking, and chronic underlying disease (including diabetes, hypertension, chronic lung, cardiovascular, kidney, or immunosuppressive disease, or other medically complex conditions).

Randomization

Patients were stratified by de novo infection and viral rebound, then underwent randomization separately. Participants were 1:1:1 assigned using a random number generated by a centralized randomization system provided by Clinical Information Management Suite (CIMS) Medical Technology Company (Chengdu, China) to 60 mg/day CEP, 120 mg/day CEO, or matching placebo group (Figure S1).

Blinding and Masking

Alibaba Health Company (Beijing, China) is responsible for drug blinding. First, a unique traceability code is generated according to the drug information. The traceability code is pasted on the corresponding test drug package and encrypted to generate the drug blinding. At the same time, it is associated with the random number of the central randomization system and randomly assigned to patients. A third party blinds the whole process. Researchers do not participate in this process.

The CIMS was responsible for generating a randomization number, and Alibaba Health Information Technology Limited was responsible for linking the randomization number to the drug and placebo and removing the manufacturer's label for masking. The three groups were blinded with 12 patients as the number of each block.

Participants were 1:1:1 assigned using a random number generated by a centralized randomization system provided by Clinical Information Management Suite (CIMS) Medical Technology Company (Chengdu, China) to 60 mg/day CEP, 120 mg/day CEP, or matching placebo group. Researchers, patients, caregivers, and statisticians were masked to allocation, and a separate unblinded data monitoring committee evaluated safety throughout this study. This study was conducted unblinding after the end of the trial.

Interventions

Participants received 40 mg of CEP, 20 mg of CEP, or matched placebo orally every 8 hours for five days (15 doses total) or until negative conversion. CEP and matching placebo were manufactured by Yun Nan Bai Yao Pharmaceutical Group Inc. (Z20026797), packaging for the matching placebo was identical to that of the associated drug. All patients at alternate care sites received standard medical treatment according to the Scheme for Diagnosis and Treatment of 2019 Novel Coronavirus Pneumonia (The 9^th^ Trial Edition) from the Health Commission of China^3^. The standard medical treatment includes:

I. Rest in bed, supportive treatment, and ensure sufficient energy and nutritional intake.

II. Pay attention to the balance of water and electrolyte to maintain homeostasis.

III. Closely monitor the vital signs of patients, especially oxygen saturation.

IV. According to the condition's needs, monitor the blood routine, urine routine, and other biochemical indicators.

V. Provide oxygen therapy according to the needs of the condition.

VI. Psychological intervention.

VII. Chinese traditional medicines such as Lianhua Qingwen capsule.

Outcome measures

The primary outcome of the efficacy was viral clearance time, the time from randomization to negative conversion (the first of two consecutive negative nasopharyngeal swabs tested by PCR, Ct value>35 for the ORF1ab and N genes). The secondary outcomes were the proportion of patients who progressed to pneumonia or severe COVID-19 and the proportion of patients who were SARS-CoV-2 positive after a negative nasopharyngeal swab.

Safety endpoints included adverse events and serious adverse events during the follow-up period. Reported adverse events were coded according to the Medical Dictionary for Regulatory Activities (MedDRA), version 25.0. The safety analysis population included all patients who received at least one dose of the study intervention drug. The incidence data for each treatment group were analyzed within the safety analysis population.

Trial Procedures

Researchers screened and confirmed eligibility at the alternate care site. Patients were stratified by de novo infection and viral rebound, then underwent randomization separately. De novo SARS-CoV-2 infected patients confirmed SARS-CoV-2 infection for the first time. Viral rebound patients had two consecutive negative PCR tests; after that, the PCR test was positive again. At screening, demographic information was collected, including eligibility criteria, medical history, concomitant medications, symptom reporting, and vaccination history of SARS-CoV-2. At randomization, we conducted a PCR test to verify the SARS-CoV-2 infection.

The central investigational pharmacy distributed the study medicine. It was combined with the central randomization system. Shipping and delivery were tracked. At the alternate care sites, nasopharyngeal swabs of included participants were collected on day 3, day 5 (after randomization) and every day after day 5 until the COVID-19 PCR swabs tested negative for two consecutive times. Adverse events and development of pneumonia or severe COVID-19 were recorded during the alternate care site visits and follow-up (until Day 28 after randomization). Patients were followed up by telemedicine visits every week after leaving the alternate care site.

Statistical Analysis

The safety analysis set included all eligible patients who received at least one dose of the study medicine. The modified intention-to-treat population (mITT) included participants who retested PCR positive at randomization and received at least one dose of the study medicine. Per-protocol-set (PPS) included participants with good medical compliance (80%-120%) and completed the trial.

Viral clearance time, the time from randomization to negative nasopharyngeal swab, was the primary endpoint. The sample size of this study was determined to detect a potential clinical superiority of CEP in time to viral shedding. According to the experimental results of CEP against the SARS-CoV-2 virus in vivo and in vitro, we supposed a total of 105 patients in each group would provide 80% power to detect a hazard ratio (HR) of 1.5. HR was evaluated by the Cox proportional hazards model of the viral clearance time for CEP over placebo. The overall probability of an event was 0.9, with a 2-sided significance level of α=0.05, and the ratio of the sample in each group was 1:1:1. Considering the explorative property of this study, and that the assumptions in sample size determination were based on limited clinical evidence, this study continued to enroll patients after 315 patients were enrolled when research resources were sufficient.

The analysis for efficacy assessment was performed in both the mITT set and the PPS. The analysis performed in the mITT set was primary analysis, and that performed in the PPS was considered supportive analysis. Continuous variables were presented as the means with standard deviations or medians with interquartile ranges (IQRs), and categorical variables were reported as numbers and percentages. There were no missing data in the mITT set.

There was no censoring for viral clearance time due to the loss of follow-up in this study. The viral clearance time was summarized, and we estimated each group's restricted mean survival time (RMST). A Cox proportional hazards model was fitted to estimate the hazard ratio (HR) and 95% confidence intervals for the 60 mg/day CEP group and 120 mg/day CEP group compared with the control group. The Cox proportional hazards model evaluated the reported p-value. In this model, underlying disease (presence or absence), age (>60 years or ≤60 years), sex (male or female), symptoms on admission (symptomatic or asymptomatic), and days from the positive PCR result to randomization were included as prespecified adjusted variables. In addition, prespecified subgroup analyses of primary and secondary endpoints were conducted, and 95% CIs were provided to evaluate whether the treatment effect varied according to age, sex, symptoms (asymptomatic or mild), or high-risk factors for progression to severe COVID-19 (including age≥60 years, smoking, obesity, and underlying clinical conditions).

Detailed reasons for patient exclusion from mITT analysis

Because the alternate care site in China was a quarantine site, before entering this area, the investigators first communicated with patients by telephone and obtained informed consent, determined the patients to be eligible for enrollment, and performed randomization. Then, the investigator in charge of medicine distribution entered the alternate care site to distribute the medicine to the patients. From June 1st, 2022, to June 3rd, 2022, the investigator in charge of medicine distribution had close contact with the COVID-19 patients because the working time at the alternate care site exceeded the specified requirements and was quarantined for three days according to the regulations at the time in China. Therefore, between June 1st, 2022, and June 3rd, 2022, 69 patients (52 de novo infected patients and 17 rebound patients) underwent randomization but did not receive the intervention medicine. In addition, among the randomized patients, 36 patients (22 de novo infected patients and 14 rebound patients) were confirmed to be SARS-CoV-2 negative by PCR at randomization.

Participant Monitoring and Data Collecting

The daily and follow-up assessments were monitored, and sites were actively notified of events requiring review, including serious adverse events. During the alternate care sites period, participants were visited daily for assessments, including any adverse events, occurrence or disappearance of symptoms, temperature, and the development of pneumonia or severe covid-19. After leaving the alternate care site, researchers followed up participants by telephone every week until day 28 after randomization. Adverse events, viral rebound, and development of Covid-19 were recorded. All data were recorded in Electronic Data Capture System provided by Blue Balloon Medical Research Co., Ltd (Beijing, China).

Cepharanthine and Placebo Information

The chemical formula of CEP is C37H38N2O6, and the molecular weight is 606.71. The CEP tablets in the study were manufactured by Yun Nan Bai Yao Pharmaceutical Group Inc (Z20026797). The appearance of the CEP tablet is white or yellowish, smells odorless, and tastes bitter. One CEP tablet contains 20mg of CEP.

Yun Nan Bai Yao pharmaceutical Group Inc supplies the placebo. The placebo is matched with CEP. The appearance is a milky white or yellowish tablet with appropriate hardness and wear resistance which has no apparent difference from the appearance of CEP tablets.

CEP and placebo should be stored at an average temperature. All packaging will be labeled to indicate that the product is for investigational use. CEP and placebo were distributed by investigators in alternate care sites.

Dosing and Administration

120mg/day CEP group: 2 CEP tablets (40mg) orally each time, three times daily for 5 days or until SARS-CoV-2 turned consecutive negative tested by PCR.

60mg/day CEP group: 1 CEP tablet (20mg) plus 1 placebo tablet orally each time, three times daily for 5 days or until SARS-CoV-2 turned consecutive negative tested by PCR.

Placebo group: 2 placebo tablets orally each time, three times daily for 5 days or until SARS-CoV-2 turned consecutive negative tested by PCR.

Study Responsibilities

Researchers took responsibility for study design and conduct, data collection and interpretation, and manuscript writing. Faculty of Statistics, Clinical Research Center, Peking University, assisted with study design, data collection and cleaning, and statistical analyses. Clinical research coordinators recorded data. Clinical research coordinators and Clinical Information Management Suite (CIMS) database personnel reviewed the collected data. The clinical research coordinators then entered confirmed data into the Clinical Information Management Suite database for statistical analyses performed by and reviewed by the senior statistician following Good Clinical Practice guidelines. Yun Nan Bai Yao Company manufactured the CEP tablets and matching placebo tablets and then completed the blinding under the guidance of a third party (Peking University Clinical Research Institute). All the data were available to all the authors, who vouch for the accuracy and completeness of the data as well as the adherence of the trial to the protocol.

**Supplementary Results**

The secondary outcome in de novo infected patients

None of the patients in the mITT set progressed to severe COVID-19 during the 28-day follow-up. Among mITT patients, the proportions of patients who were SARS-CoV-2 positive after negative nasopharyngeal swabs in the 120 mg/day CEP, 60 mg/day CEP, and placebo groups were 3.1% (2/65), 1.5% (1/68) and 5.5% (3/55), respectively [Supplementary materials Table S3].

Results in viral rebound patients

Between May 31st and July 24th, 2022, 551 patients were screened for inclusion at two sites in Shanghai, China; 262 de novo SARS-CoV-2 infected patients and 124 viral rebound patients underwent randomization. There were 93 viral rebound patients included in the mITT set. Viral rebound patients in the mITT set were allocated to the 120 mg/day CEP (n=29), 60 mg/day CEP (n=34), and placebo groups (n=30). The patients included in the PPS in the three groups were 29, 33, and 28, respectively. All patients in the mITT set had completed the 28-day follow-up [Figure S1 b].

Among viral rebound patients in the mITT set, the viral clearance time in the 120 mg/day CEP, 60 mg/day CEP, and placebo groups was 2.45 (95% CI 1.97 to 2.93), 2.41 (95% CI 2.13 to 2.70) and 2.37 (95% CI 2.07 to 2.67) days, respectively. Compared with placebo, neither 120 mg/day of CEP nor 60 mg/day of CEP significantly shortened the time. Further analysis of the PPS also showed no significant difference in the viral clearance time between the CEP treatment groups and the placebo group [Table S4].

**Supplementary References**

1 Gandhi, R. T., Lynch, J. B. & Del Rio, C. Mild or Moderate Covid-19. *The New England Journal of Medicine* **383**, 1757-1766, doi:10.1056/NEJMcp2009249 (2020).

2 Berlin, D. A., Gulick, R. M. & Martinez, F. J. Severe Covid-19. *The New England Journal of Medicine* **383**, 2451-2460, doi:10.1056/NEJMcp2009575 (2020).

3 China, N. H. C. o. t. P. s. R. o. Chinese guideline for the management covid-19 (version 9.0, in Chinese). <http://www.nhc.gov.cn/yzygj/s7653p/202203/b74ade1ba4494583805a3d2e40093d88/files/ef09aa4070244620b010951b088b8a27.pdf> (2022).

| **Table S1. Demographic and Clinical Characteristics of the De Novo Infected Patients (mITT population)** | | | | |
| --- | --- | --- | --- | --- |
|  | **No.(%)** |  |  |  |
| **Characteristic** | **Overall** | **120mg/day CEP** | **60mg/day CEP** | **Placebo** |
| **No.** | 188 | 65 | 68 | 55 |
| **Age, median (IQR), y** | 40.00 [29.00, 52.25] | 41.00 [31.00, 54.00] | 35.50 [26.50, 49.25] | 43.00 [31.50, 51.50] |
| **>60 y (%)** | 21 (11.2) | 9 (13.8) | 7 (10.3) | 5 (9.1) |
| **Sex (%)** |  |  |  |  |
| **female** | 74 (39.4) | 20 (30.8) | 34 (50.0) | 20 (36.4) |
| **male** | 114 (60.6) | 45 (69.2) | 34 (50.0) | 35 (63.6) |
| **Symptom type of patients (%)** |  |  |  |  |
| **Symptomatic** | 119 (63.3) | 44 (67.7) | 42 (61.8) | 33 (60.0) |
| **Had symptoms at enrolment** | 52 (27.7) | 19 (29.2) | 16 (23.5) | 17 (30.9) |
| **Fever** | 5 (2.7) | 3 (4.6) | 0 (0.0) | 2 (3.6) |
| **Cough** | 48 (25.5) | 17 (26.2) | 16 (23.5) | 15 (27.3) |
| **Asymptomatic** | 69 (36.7) | 21 (32.3) | 26 (38.2) | 22 (40.0) |
| **Days from first nucleic acid test to randomization (%)** | |  |  |  |
| **≤ 3 days** | 101 (53.7) | 33 (50.8) | 39 (57.4) | 29 (52.7) |
| **4-5 days** | 87 (46.3) | 32 (49.2) | 29 (42.6) | 26 (47.3) |
| **Vaccine (%)** |  |  |  |  |
| **Not vaccinated** | 32 (17.0) | 8 (12.3) | 14 (20.6) | 10 (18.2) |
| **Vaccinated, 1-2 doses** | 67(35.7%) | 18 (27.7) | 28 (41.2) | 21 (38.2) |
| **Vaccinated, 3 doses** | 89 (47.3) | 39 (60.0) | 26 (38.2) | 24 (43.6) |
| **Patients at high risk of developing severe COVID-19 (%)*** | 98 (52.4) | 35 (53.8) | 36 (53.7) | 27 (49.1) |
| **With underlying chronic disease** | 36 (19.1) | 13 (20.0) | 14 (20.6) | 9 (16.4) |
| **Age > 60 y** | 21 (11.2) | 9 (13.8) | 7 (10.3) | 5 (9.1) |
| **BMI > 25 kg/m^2^** | 47 (25.1) | 16 (24.6) | 17 (25.4) | 14 (25.5) |
| **Cigarette smoking** | 50 (26.6) | 18 (27.7) | 18 (26.5) | 14 (25.5) |
| ***Patients at risk of developing severe COVID-19 were defined as have ≥1 of following: >60 year of age; chronic underlying disease; BMI>25 kg/m^2^; cigarette smoking** | | | | |

| **Table S2. Demographic and Clinical Characteristics of the Patients (viral rebound patients, mITT)** | | | |  |
| --- | --- | --- | --- | --- |
|  | **No.(%)** |  |  |  |
| **Characteristic** | **120mg/day CEP** | **60mg/day CEP** | **Placebo** | **P value** |
| **No.** | 29 | 34 | 30 |  |
| **Age, median (IQR), y** | 55.00 [46.00, 62.00] | 48.00 [36.00, 55.00] | 44.50 [35.25, 57.50] | 0.088 |
| **>60 y (%)** | 9 (31.0) | 3 (8.8) | 6 (20.0) | 0.091 |
| **Sex (%)** |  |  |  | 0.528 |
| **female** | 6 (20.7) | 8 (23.5) | 10 (33.3) |  |
| **male** | 23 (79.3) | 26 (76.5) | 20 (66.7) |  |
| **Symptom type of patients (%)** |  |  |  |  |
| **Symptomatic** | 3 (10.3) | 4 (11.8) | 2 (6.7) | 0.781 |
| **Had symptoms at enrolment** | 1 (3.4) | 3 (8.8) | 1 (3.3) | 0.617 |
| **Fever** | 1 (3.4) | 2 (5.9) | 0 (0.0) | 0.642 |
| **Cough** | 0 (0.0) | 2 (5.9) | 1 (3.3) | 0.772 |
| **Asymptomatic** | 26 (89.7) | 30 (88.2) | 28 (93.3) |  |
| **Days from first nucleic acid test to randomization (%)** |  |  |  | 0.337 |
| **≤ 3 days** | 28 (96.6) | 29 (85.3) | 28 (93.3) |  |
| **4-5 days** | 1 (3.4) | 5 (14.7) | 2 (6.7) |  |
| **Vaccine (%)** |  |  |  | 0.066 |
| **Not vaccinated** | 10 (34.5) | 4 (11.8) | 13 (43.3) |  |
| **Vaccinated, 1-2 doses** | 5 (17.2) | 8 (23.5) | 5 (16.7) |  |
| **Vaccinated, 3 doses** | 14 (48.3) | 22 (64.7) | 12 (40.0) |  |
| **Patients at high risk of developing severe COVID-19 (%)*** | 18 (62.1) | 25 (73.5) | 20 (66.7) | 0.62 |
| **With underlying chronic disease** | 8 (27.6) | 8 (23.5) | 10 (33.3) |  |
| **Age > 60 y** | 9 (31.0) | 3 (8.8) | 6 (20.0) |  |
| **BMI > 25 kg/m^2^** | 10 (34.5) | 13 (38.2) | 5 (16.7) |  |
| **Cigarette smoking** | 9 (31.0) | 11 (32.4) | 10 (33.3) |  |
| ***Patients at risk of developing severe COVID-19 were defined as have ≥1 of following: >60 year of age; chronic underlying disease; BMI>25 kg/m^2^; cigarette smoking** | | | |  |

| **Table S3 Secondary outcomes in three groups of mITT population. (28 Days)** | | | |
| --- | --- | --- | --- |
|  | **120 mg/day CEP** | **60 mg/day CEP** | **Placebo** |
| **De novo infected patients** |  |  |  |
| No. | 65 | 68 | 55 |
| Patients developed to pneumonia or severe COVID-19 — no.(%) | 0(0) | 0(0) | 0(0) |
| SARS-CoV-2 returned to positive after turning negative — no.(%) | 2 (3.1) | 1 (1.5) | 3 (5.5) |
| Hospitalization for COVID-19 — no.(%) | 0(0) | 0(0) | 0(0) |
| **Viral rebound patients** |  |  |  |
| No. | 29 | 34 | 30 |
| Patients developed to pneumonia or severe COVID-19 — no.(%) | 0(0) | 0(0) | 0(0) |
| SARS-CoV-2 returned to positive after turning negative — no.(%) | 1(3.4) | 0(0) | 1(3.3) |
| Hospitalization for COVID-19 — no.(%) | 0(0) | 0(0) | 0(0) |

| **Table S4. Viral clearance time in three groups (viral rebound patients).** | | | | | | |
| --- | --- | --- | --- | --- | --- | --- |
|  | **mITT** | | | **PPS** | | |
|  | **120mg/day CEP** | **60mg/day CEP** | **Placebo** | **120mg/day CEP** | **60mg/day CEP** | **Placebo** |
| **N (missing)** | 29 (0) | 34 (0) | 30 (0) | 29 (0) | 33 (0) | 28 (0) |
| **Outcome event (%)** | 29 (100) | 34 (100) | 30 (100) | 29 (100) | 33 (100) | 28 (100) |
| **Viral clearance time *(95%CI), days** | 2.45  (1.97, 2.93) | 2.41  (2.13, 2.70) | 2.37  (2.07, 2.67) | 2.45  (1.96, 2.93) | 2.39  (2.10, 2.68) | 2.25  (2.06, 2.44) |
| **Difference (95%CI)** | 0.08  (-0.49, 0.65) | 0.05  (-0.37, 0.46) | - | 0.20  (-0.32, 0.72) | 0.14  (-0.20, 0.49) | - |
| **Hazard Ratio**  **(95%CI)** | 1.02  (0.59, 1.77) | 0.85  (0.51, 1.42) | - | 0.97  (0.56, 1.70) | 0.79  (0.46, 1.4) | - |
| **P value**** | 0.941 | 0.534 | - | 0.928 | 0.382 | - |
| *Viral clearance time was the time from randomization to negative nasopharyngeal swab. RMST evaluated it.  **p value was evaluated by Cox proportional hazards model, adjusted for de novo or viral rebound, underlying chronic disease, age (>60 yr or ≤60 yr), gender, symptoms, days from first nucleic acid test to randomization. | | | | | | |

| **Table S5 Subgroup analysis of patients whether have Chinese traditional medicine** | | | | | | | |
| --- | --- | --- | --- | --- | --- | --- | --- |
| **Chinese traditional medicine** | **no. of events** | | | **60mg/day vs. placebo** | | **120mg/day vs. placebo** | |
|  | 60 mg/day | 120 mg/day | placebo | Hazard Ratio (95%CI) | p-value | Hazard Ratio (95%CI) | p-value |
| yes | 44 | 50 | 40 | 1.43(0.91,2.25) | 0.125 | 1.20(0.77,1.86) | 0.4232 |
| no | 24 | 15 | 15 | 0.90(0.41,1.95) | 0.785 | 1.01(0.43,2.34) | 0.988 |


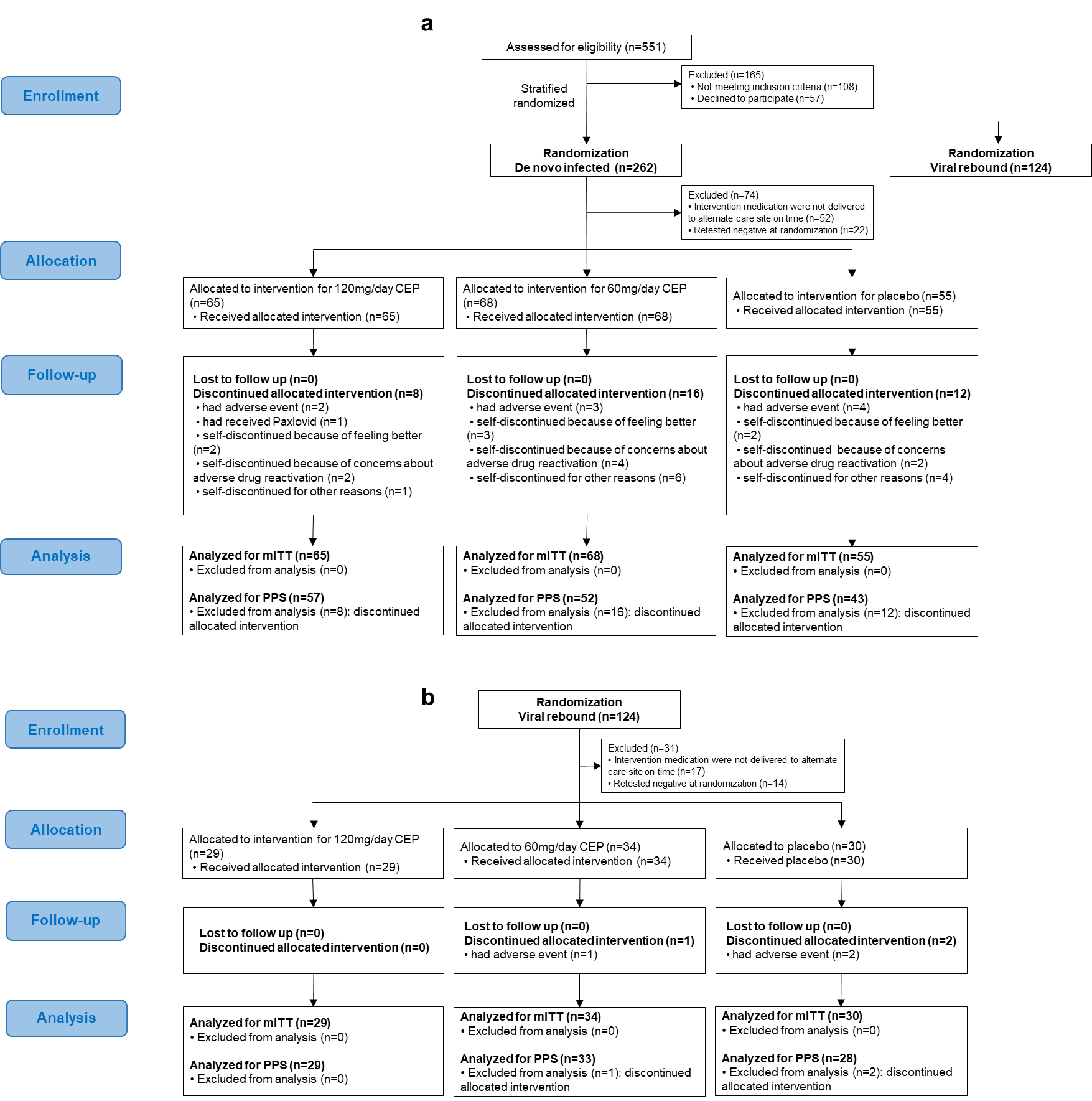
Figure S1

**Figure S1.** **Randomization, treatment assignments, and follow-up.**

**a,b** The figure shows that patients were recruited from May 31st, 2022, to July 24th, 2022, from two alternate care sites in Shanghai, China, and underwent stratified randomization according to de novo infection or viral rebound of SARS-CoV-2. Treatment assignments and follow-up were conducted.


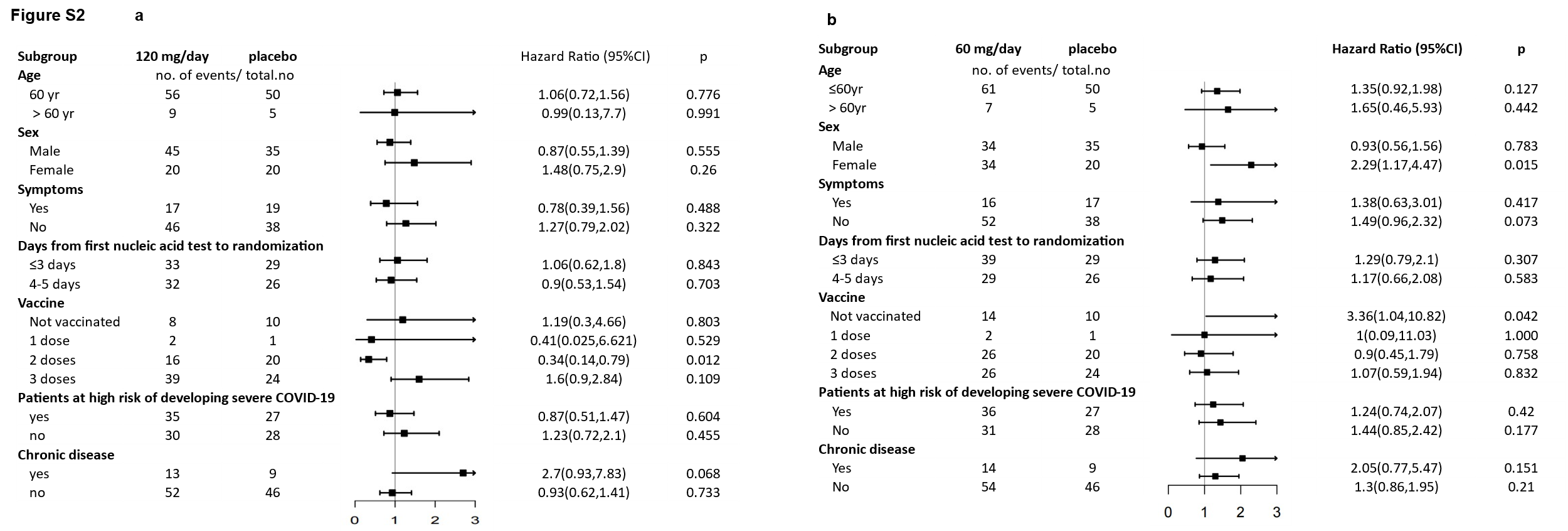
Figure S2

**Figure S2. Subgroup analysis of the difference in the viral clearance time compared to placebo in the de novo infected patients (mITT).**

**a** Subgroup analysis of patients who received 120 mg/day of CEP and those who received placebo. **b** Subgroup analysis between patients who received 60 mg/day of CEP and those who received placebo.

Safety and Efficacy of Oral Administrated Cepharathine in Non-hospitalized, Asymptomatic or Mild COVID-19 Patients: A Double-blind, Randomized, Placebo-controlled Trial

Statistical Analysis Plan

(SAP)

Version: V1.0

Date: July 8^th^, 2022

By Peking University Clinical Research Institute

**1. Title**

Safety and Efficacy of Oral Administrated Cepharathine in Non-hospitalized, Asymptomatic or Mild COVID-19 Patients: A Double-blind, Randomized, Placebo-controlled Trial

**2. Objective**

Evaluate in the adult patients infected with SARS-CoV-2 (both de novo infected patients and viral rebound patients) based on standard medical treatment, compared with placebo, whether the Cepharanthine (CEP) Tablets (Z20026798) can effectively shorten the viral clearance time.

**3. Study design**

**3.1. General description of the study**

This project is a prospective, randomized, double-blind, placebo-controlled three-arm intervention study initiated by researchers (IIT). Due to the epidemic situation, the research is expected to conduct from May 2022 to July 2022.

Treatment group (low dose of CEP): standard medical treatment and 20 mg CEP tid for 5 days.

Treatment group (high dose of CEP): standard medical treatment and 40 mg CEP tid for 5 days.

Control group: standard medical treatment and matched placebo for 5 days.

**3.2. Sample size**

According to the average overcast days of Shanghai SARS-CoV-2 Omicron mutant is 11.13 days, it is assumed that CEP tablets can shorten the overcast days. According to Cox regression calculation, compared with the control group, the number of days when the virus turns negative is shortened, Hazard Ratio, HR=2.0, with bilateral 95% Confidence interval, obtaining unilateral p<0.05 is the sample size calculation method, and the sample size of the treatment group needs 73 cases. Set HR=1.7 106 cases are needed. HR=1.5, 210 cases are needed. The sample size of this study was determined to detect a potential clinical superiority of CEP in time to viral shedding. According to the experimental results of CEP against the SARS-CoV-2 virus in vivo and in vitro, we supposed a total of 105 patients in each group would provide 80% power to detect an HR of 1.5, which was tested in the Cox proportional hazards model of the time to negative nasopharyngeal swab for CEP over placebo. The overall probability of an event was 0.9, with a 2-sided significance level of α=0.05, and the ratio of the sample in each group was 1:1:1. Considering the explorative property of this study, and that the assumptions in sample size determination were based on limited clinical evidence, this study continued to enroll patients after 315 patients were enrolled when research resources were sufficient.


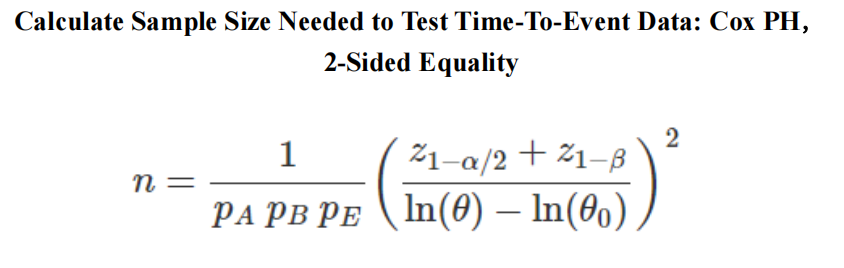


θ0 is the hazard ratio hypothesized under the null hypothesis

θ is the hazard ratio

ln(θ) is the natural logarithm of the hazard ratio, or the log-hazard ratio

pE is the overall probability of the event occurring within the study period

pA and pB are the proportions of the sample size allotted to the two groups, named 'A' and 'B'

**3.3. Randomization and blinding**

Central randomization (CIMS random system).

The three groups were blinded with 12 cases as the number of people in each block. Researchers, subjects, nurses, and data analysts are all blinded.

Medication distribution: three groups in total, high dose group (2 tablets of 20 mg of CEP), low dose group (1 tablet of 20 mg of CEP and 1 tablet of placebo), placebo group (2 placebo tablets)

Blinding of drugs: the pharmaceutical factory carries out the non-difference outer packaging of drugs and placebo in the GMP workshop, and under the guidance of a third party (Peking University clinical research), the pharmaceutical factory completed the blinding of drugs and placebo.

**3.4. Population**

1) Adult patients with asymptomatic or mild SARS-CoV-2 de novo infection.

2) Adult patients with asymptomatic or mild SARS-CoV-2 viral rebound.

**3.5 Dose of medication**

de novo SARS-CoV-2 infection group:

1. Low dose of CEP: SMT + oral CEP tablet (3 times/day, 20 mg each time) for 5 days.

2. High dose of CEP: SMT + oral CEP tablet (3 times/day, 40 mg each time) for 5 days.

3. Placebo group: SMT + matched placebo.

SARS-CoV-2 viral rebound group:

1. Low dose of CEP: SMT + oral CEP tablet (3 times/day, 20 mg each time) for 5 days.

2. High dose of CEP: SMT + oral CEP tablet (3 times/day, 40 mg each time) for 5 days.

3. Placebo group: SMT + matched placebo.

**4. Criteria**

Inclusion Criteria:

1) aged over 16 years old with all genders

2) SARS-CoV-2 positive（laboratory-confirmed reverse transcription polymerase chain reaction (RT PCR) test)

3) patient or immediate adult family member agrees to participate in this study and signs an informed consent form

4) with mild covid-19 symptoms

5) confirmed SARS-CoV-2 infection within 5 days prior to randomization

Exclusion Criteria:

1) Confirmed SARS-CoV-2 infection within > 5 days prior to randomization

2) With pneumonia on admission

3) diagnosed as severe or critical COVID-19 before intervention

4) has a history of chronic underlying disease and acute exacerbation of that underlying disease at the time of admission

5) Females who are pregnant or breastfeeding

**4. Flow diagram**

**4.1 Flow diagram of study**


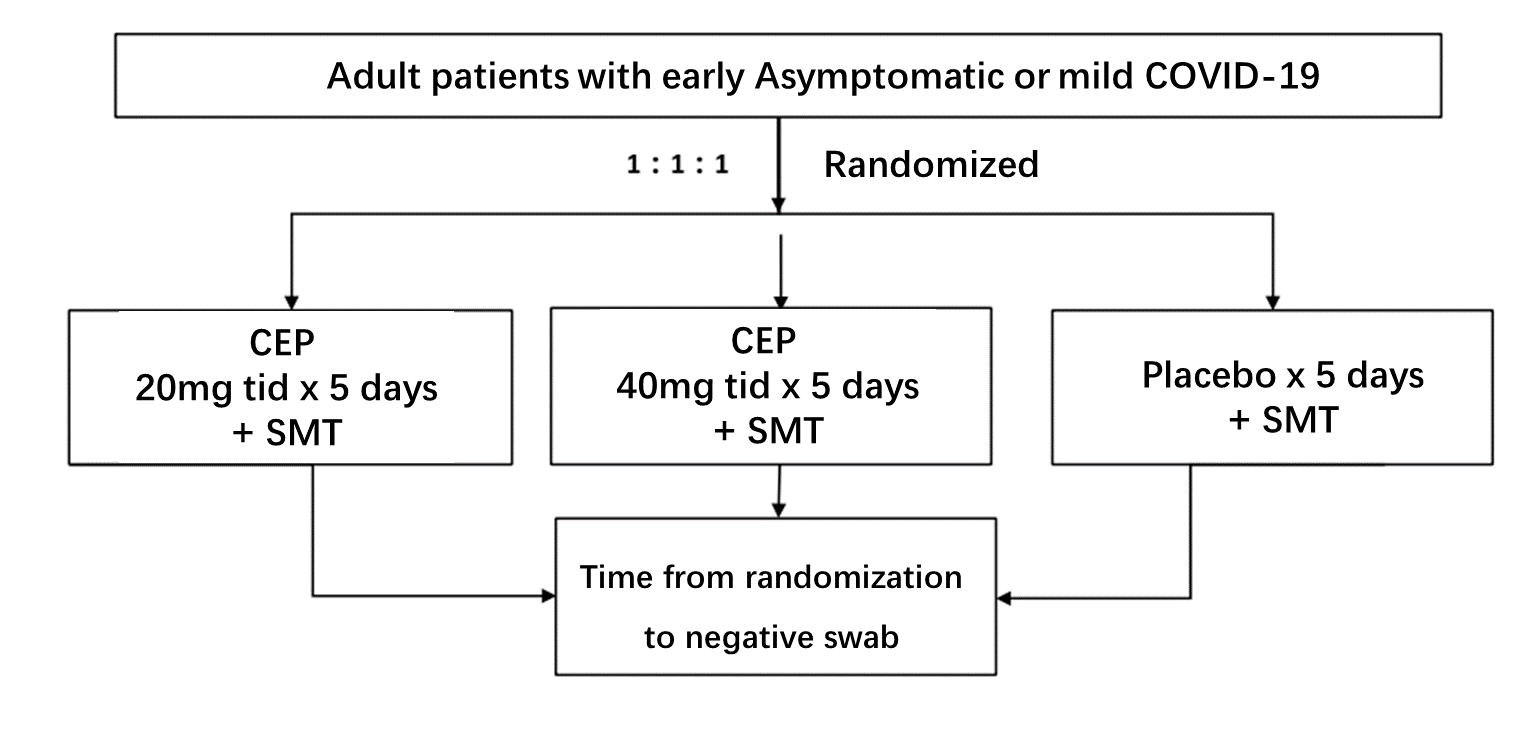


**4.2 Flow chart of patients observation and follow-up**


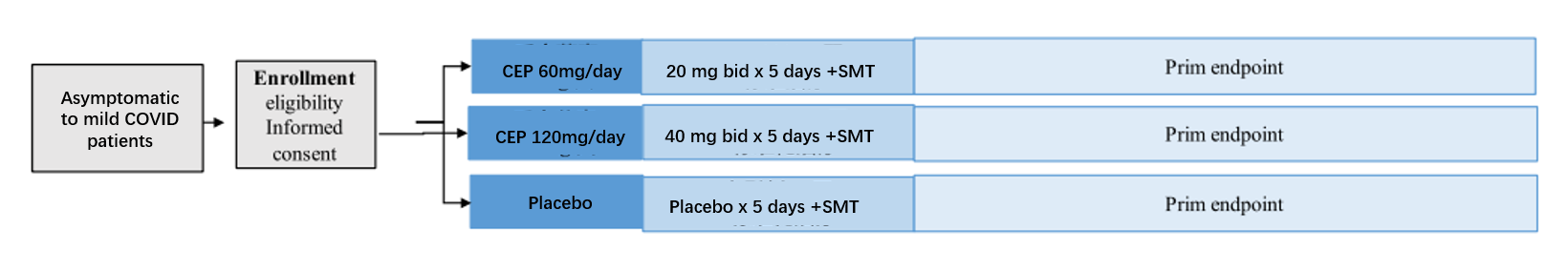


**5. Efficacy and safety**

5.1 Efficacy

5.1.1 Primary endpoint: Viral clearance time, the time from randomization to the negative time. Negative time was the first of two consecutive negative nasopharyngeal swabs tested by PCR, Ct value>35 for the ORF1ab and N genes.

5.1.2 Secondary endpoints:

(1) Proportion of participants developing COVID-19 pneumonia

(2) Proportion of participants developing severe pneumonia

(3) Proportion of patients who were SARS-CoV-2 positive after a negative nasopharyngeal swab.

(4) Number of days from the onset of fever until the temperature drops below 37.3°C

5.2 Safety

Incidence of Adverse Events (AEs) and Serious Adverse Events (SAEs).

**6. Statistical analysis**

**6.1. Statistical analysis data set**

Safety Analysis Set (SS): To analyze the adverse events. Including participants (de novo infected patients and rebound patients) who received at least 1 dose of CEP or placebo.

Modified Intention To Treat (MITT): It is the dataset obtained after the least and most fair exclusion of participants from all randomized population, including participants who have received at least one study medicine after enrollment. Participants with PCR tested negative (Ct >35) at randomization should be excluded.

Per-protocol set (PPS): It is a subset of the MITT that complies more with the protocol. Participants in the PPS should have good medication compliance (>80%), no missing main efficacy indicators, and complete the trial

MITT and PPS will be used for efficacy analysis.

**6.2. Statistical content and methods**

SAS9.4 statistical analysis software and R will be used for statistical analysis.

All statistical tests are double-sided (unless otherwise specified), and a P value less than or equal to 0.05 will be considered statistically significant.

6.2.1. Completion and population analysis

Summarize the screening, enrollment, and Completion numbers of each center and stratified factor, and list the shedding cases. Determine the size of the analysis data set, the case distribution of each center and each stratified factor, the comparison of the total dropout rate, and the detailed list of unfinished reasons.

The demographic characteristics (age, height, vital signs, etc.), medical history, SARS-Cov-2 infection history, smoking history, allergy history, and medication history of the patients were described, and the age, height, weight, and baseline vital signs of the two groups were compared to measure the comparability between the groups.

6.2.2. Medical compliance evaluation

Compliance evaluation (%)=actual medication quantity/theoretical medication quantity × 100%；

Good compliance: 80% - 120%;

Poor compliance: ＜ 80% or ＞ 120%.

Descriptive statistics were made on compliance (%) according to quantitative indicators, and descriptive statistics were made on compliance good/bad according to classification indicators.

6.2.3. Main efficacy index: viral clearance time

Viral clearance time was the time from randomization to negative swab (the first time of two consecutive negative PCR tests, Ct value ≤ 35). Because patients were isolated in the alternate care sites until their PCR tests turned negative, all patients included in this study could be observed the outcome event. Participants who did not have negative PCR tests during the follow-up would be censored. Kaplan-Meier curve was drawn for the time of nucleic acid turning negative in each group. Log-rank test was used to compare the survival curve between groups. Cox regression model was established with PCR negative as the end point event, and the independent variables included group, center, gender, age, etc. The HR and 95% confidence interval of the low dose group and the high dose group were calculated respectively compared with the placebo control group.

In addition, the restricted mean survival time (RMST) of each group will be calculated, and the difference and 95% confidence interval of RMST between the low dose group and the high dose group of CEP and the placebo control group will be calculated respectively. Prespecified subgroup analyses of primary and secondary endpoints were conducted, and 95% CIs were provided to evaluate whether the treatment effect varied according to age, sex, symptoms (asymptomatic or mild), or high-risk factors for progression to severe COVID-19 (including age≥60 years, smoking, obesity, and underlying clinical conditions).

6.2.4. Secondary efficacy indicators

(1) Proportion of asymptomatic progression to COVID-19 during hospitalization

According to qualitative indicators, descriptive statistics and inter group comparison were made on the proportion of patients in each group who developed from asymptomatic to COVID-19 during hospitalization, and the rate difference and 95% confidence interval between the low-dose group and high-dose group of CEP and the placebo control group were calculated respectively.

(2) Proportion of severe pneumonia progressing from asymptomatic during hospitalization

According to qualitative indicators, descriptive statistics and inter-group comparison were made on the proportion of severe pneumonia from asymptomatic progression during hospitalization in each group, and the rate difference and 95% confidence interval between the low dose group and the high dose group of CEP and the placebo control group were calculated respectively.

(3) Proportion of patients who had two consecutive negative PCR tests and discharged, after that, those patients had a positive PCR test again with 14 days.

According to qualitative indicators, descriptive statistics and inter-group comparison were performed in each group, and the rate difference and 95% confidence interval were calculated respectively in the low-dose and high-dose CEP tablets group compared with the placebo control group.

(4) Days from fever to temperature falling below 37.3 ° C

According to quantitative indicators, descriptive statistics and inter-group comparison were made on the days from fever to temperature falling below 37.3 ° C in each group, and the difference and 95% confidence interval between the low dose group and the high dose group of CEP and the placebo control group were calculated respectively.

**6.3.5. Safety index evaluation**

Safety analysis will be conducted for the safety analysis population (SS population).

Adverse events (AEs)

Calculate the number and proportion of adverse events and serious adverse events in each group. In addition, the above events will be summarized according to the severity and the relationship with the intake of study drugs.
